# Inflammation-related collagen fibril destruction contributes to temporomandibular joint disc displacement via NF-κB activation

**DOI:** 10.1038/s41368-025-00352-0

**Published:** 2025-04-17

**Authors:** Shengjie Cui, Yanning Guo, Yu Fu, Ting Zhang, Jieni Zhang, Yehua Gan, Yanheng Zhou, Yan Gu, Eileen Gentleman, Yan Liu, Xuedong Wang

**Affiliations:** 1 Department of Orthodontics, Peking University School and Hospital of Stomatology & National Center for Stomatology & National Clinical Research Center for Oral Diseases & National Engineering Laboratory for Digital and Material Technology of Stomatology & Beijing Key Laboratory of Digital Stomatology & Research Center of Engineering and Technology for Computerized Dentistry Ministry of Health & NMPA Key Laboratory for Dental Materials, Beijing, China; 2 Department of General Dentistry, Peking University School and Hospital of Stomatology, Beijing, China; 3 Fourth Clinical Division, Peking University School and Hospital of Stomatology, Beijing, China; 4 Center for Temporomandibular Disorders and Orofacial Pain, Peking University School and Hospital of Stomatology, Beijing, China; 5 Centre for Craniofacial and Regenerative Biology, King’s College London, London, UK; 6 Central Laboratory, Peking University School and Hospital of Stomatology, Beijing, China

**Keywords:** Oral diseases, Mechanisms of disease

## Abstract

Temporomandibular joint (TMJ) disc displacement is one of the most significant subtypes of temporomandibular joint disorders, but its etiology and mechanism are poorly understood. In this study, we elucidated the mechanisms by which destruction of inflamed collagen fibrils induces alterations in the mechanical properties and positioning of the TMJ disc. By constructing a rat model of TMJ arthritis, we observed anteriorly dislocated TMJ discs with aggravated deformity in vivo from five weeks to six months after a local injection of Freund’s complete adjuvant. By mimicking inflammatory conditions with interleukin-1 beta in vitro, we observed enhanced expression of collagen-synthesis markers in primary TMJ disc cells cultured in a conventional two-dimensional environment. In contrast, three-dimensional (3D)-cultivated disc cell sheets demonstrated the disordered assembly of inflamed collagen fibrils, inappropriate arrangement, and decreased Young’s modulus. Mechanistically, inflammation-related activation of the nuclear factor kappa-B (NF-κB) pathway occurs during the progression of TMJ arthritis. NF-κB inhibition reduced the collagen fibril destruction in the inflamed disc cell sheets in vitro, and early NF-κB blockade alleviated collagen degeneration and dislocation of the TMJ discs in vivo. Therefore, the NF-κB pathway participates in the collagen remodeling in inflamed TMJ discs, offering a potential therapeutic target for disc displacement.

## Introduction

The temporomandibular joint (TMJ) disc is composed of fibrocartilaginous tissue and plays a central role in mandibular movements.^1^ Disc dysfunction, including disc displacement with or without reduction, is common in the general population.^2^ The occurrence of disc displacement without reduction significantly affects patient quality of life,^3^ often resulting in abnormal bone remodeling of the condyle,^4^ orofacial pain and limited mandibular mobility.^5^ Increased levels of proinflammatory cytokines have been detected in the synovial fluid and local tissue of patients with disc dysfunction.^6,7^ Clinical studies suggest that deformation of the TMJ disc is associated with sustained inflammation and disc displacement;^8,9^ meanwhile, previous studies have elucidated the tight relationship between inflammatory stimulation with the expression levels of collagen proteins.^10,11^ However, they fall short in revealing the progression of three-dimensional (3D) structural destruction of the collagen network caused by inflammation, and the pathogenic process leading to disc dysfunction under inflammatory conditions.

Collagen and proteoglycan networks constitute the TMJ disc, and excessive degradation of these extracellular matrix (ECM) is closely associated to local inflammation and disc dysfunction.^12^ In particularly, collagen plays a vital role in maintaining the normal shape of TMJ disc and dispersing the stress generated during mastication.^13^ We previously showed that chronic inflammation in the TMJ resulted in disc deformation, accompanied by increased cell numbers and collagen content,^14^ along with deterioration of the ultrastructure and nanomechanical properties of the TMJ disc.^15,16^ To date, it is not clear how inflammation influences collagen remodeling, architecture, and its ability to buffer pressure on the TMJ disc. Therefore, elucidating the effects of inflammation on the collagen network that comprises the TMJ disc may serve as a therapeutic target to prevent degenerative changes and disc dysfunction.

Interleukin-1 beta (IL-1β) is one of the most important cytokines in the progression of inflammation.^17,18^ Previous research has suggested that exogenous IL-1β stimulation induces inflammatory responses and matrix degradation in TMJ-resident cells through activation of the nuclear factor-kappa B (NF-κB) pathway.^19–21^ Therefore, blocking NF-κB activation in TMJ disc cells may be a novel approach to reduce inflammation-related collagen matrix degeneration in TMJ discs. However, the potential therapeutic effects of NF-κB blockade have not yet been examined beyond the molecular biology level.

In the present study, we explored the biological mechanisms underlying TMJ disc displacement related to chronic inflammation, focusing on the signaling pathways that influence the remodeling of the collagen network. By creating a rat model of TMJ arthritis, magnetic resonance imaging (MRI) was applied to evaluate disc configuration and location. Additionally, we also established an in vitro model to study collagen fibril assembly and network architecture in TMJ disc cell sheets. Our findings demonstrated that sustained IL-1β stimulation leads to disorganized assembly of newly-formed collagen microfibrils with decreased mechanical properties. Mechanistically, IL-1β stimulation induced overexpression of collagen type I (COL I) and deterioration of collagen architecture via NF-κB activation. In our rat model, blocking the NF-κB pathway effectively alleviated degenerative changes in the collagen network of the TMJ disc under prolonged inflammatory conditions, ultimately preventing disc displacement. These findings may contribute to the design of molecular therapies for patients with temporomandibular disorders.

## Results

### Articular disc deformation and dislocation with increased IL-1β expression in a rat TMJ arthritis model

**Fig. 1:**
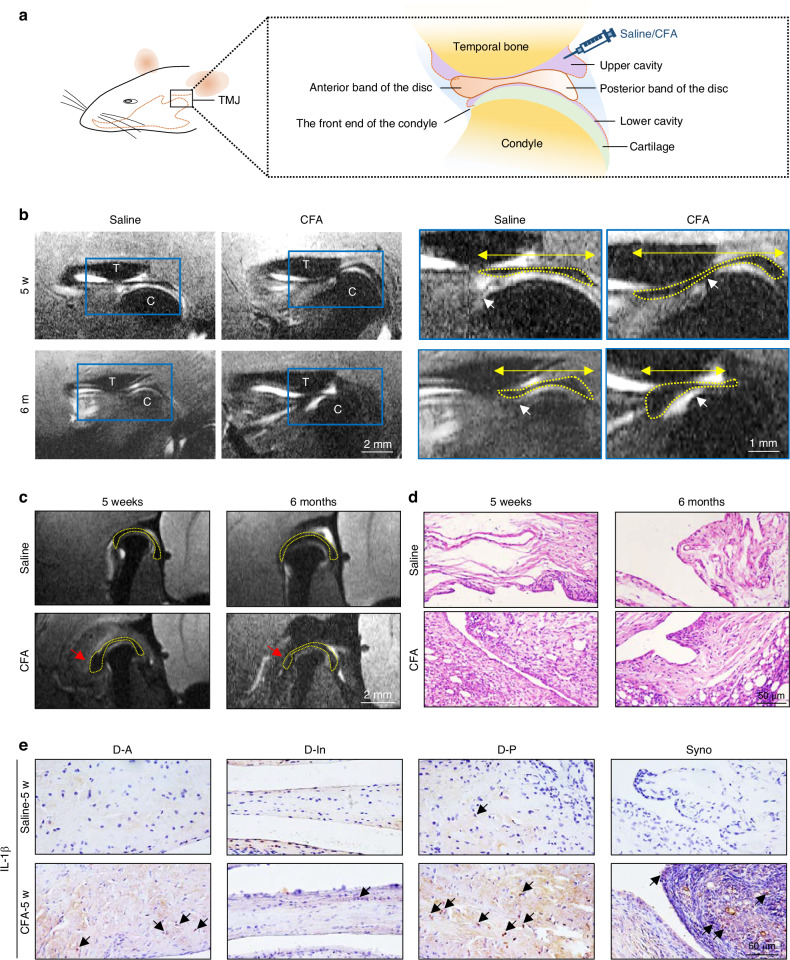
Chronic inflammation induces deformation and displacement of rat TMJ discs. **a** Anatomical diagram of the temporomandibular joint in rats subjected to the CFA intra-articular injection model. **b** Representative sagittal view of T_2_ weighted-MRI images of the rat head at 5 weeks (w) and 6 months (m) after saline/1^st^ CFA injection: TMJ discs from the rats in the control group showed biconcave contours, whereas those from rats in the inflammation-induced group were elongated at 5 w and severely deformed at 6 m, both with a dislocated anterior section (*n* = 3). T: temporal bone; C: condyle; white arrows: the most anterior aspect of the condyle; yellow line with arrows: distance between the margin of the anterior band and the posterior band of the discs; yellow dotted line: disc contour. **c** Coronal view of T_2_ weighted-MRI images of the rat head at 5 w and 6 m after saline/CFA 1^st^ injection. The control disc was located on the condyle with hypo intensity, and the upper cavity showed hyper intensity between disc and temporal bone. The lateral band in inflamed disc was thickened and folded (red arrow). yellow dotted line: disc contour. **d** Representative images of H&E staining of synovial tissue of normal and inflamed TMJ discs at 5 w and 6 m timepoint (*n* = 3 or 4). **e** Representative images of immunohistochemical staining of IL-1β of normal and inflamed TMJ discs and synovial tissue (*n* = 4). D: disc, Syno: synovial tissue, A: anterior band; In: intermediate zone, P: posterior band. Black arrows: positively stained cells

To observe changes in TMJ discs, we established a rat TMJ arthritis model by giving 2 intra-articular injections of Freund’s complete adjuvant (CFA) 14 days apart (Fig. 1a). MRI analysis revealed that the control TMJ discs had biconcave contours such that the anterior edge of the disc was opposite to the most anterior aspect of the condyle in the sagittal view (Fig. 1b). After the induction of chronic inflammation, the TMJ disc took an elongated shape with the front edge significantly extending around 30% beyond the front end of the condyle at 5 weeks after injection (Fig. S1), indicating anterior dislocation of the TMJ disc; and the disc occurred severely deformity after 6 months, accompanied with anteriorly dislocation of around 45% ahead of the condyle. Meanwhile, the lateral band of the inflamed disc was located outside the outermost edge of the condyle (Fig. 1c), indicating lateral displacement of the disc. Hematoxylin and eosin (H&E) staining of the TMJ synovial tissue demonstrated sustained inflammatory responses, with infiltrating immune cells and synovial lining cells arranged in multi-layers until 6 months after CFA local injection (Fig. 1d). Immunohistochemical staining revealed high expression of IL-1β, a key inflammatory cytokine associated with temporomandibular disorders (TMD),^22^ in the both discs and synovial tissue of CFA-injected rats (Fig. 1e). Additionally, the expression of tumor necrotic factor-alpha (TNF-α) in synovial tissue was also elevated (Fig. S2). These findings confirm that intra-articular injection of CFA induces chronic inflammation in rat TMJ, which is associated with increased IL-1β expression and leads to TMJ disc deformation and displacement.

### IL-1β promotes proliferation and collagen synthesis abilities of disc cells

**Fig. 2:**
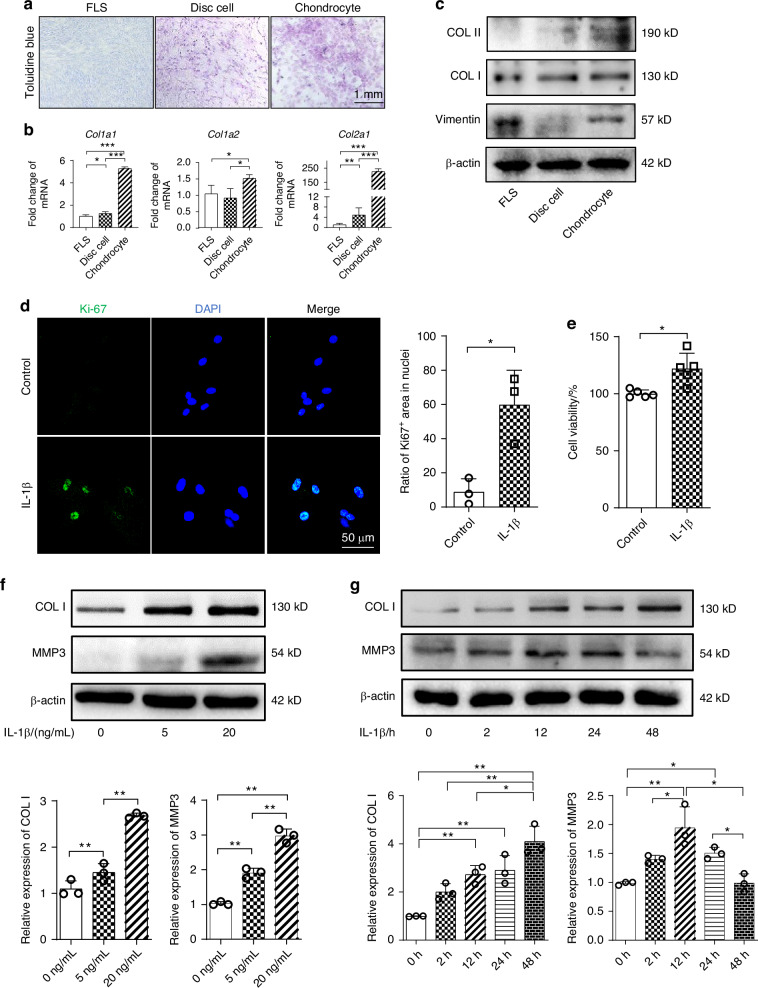
IL-1β stimulation enhances cell proliferation and collagen synthesis abilities of disc cells. **a** The disc cells showed positive toluidine blue staining compared with FLS, but the staining was weaker than chondrocytes. FLS: fibroblast-like synoviocyte. **b** The expression of *Col1a1* and *Col1a2* mRNA in disc cells was comparable with FLS, and the expression of *Col2a1* mRNA in disc cells significantly upregulated compared with FLS but significantly downregulated compared with chondrocytes. **c** The results of western blot showed that disc cells had higher expression of COL II and downregulation of vimentin compared with FLSs and no significant changes of COL I among the TMJ-derived cells. **d** Representative immunofluorescence images and semi-quantitative statistics of Ki-67 staining of disc cells stimulated with IL-1β for 24 h. **e** TMJ disc cell viability upon IL-1β stimulation over 24 h as assessed by the CCK8. **f**, **g** Expression of MMP3 and COL I assessed by western blot after IL-1β treatment at different concentrations (**f**) and timepoints (**g**). *P < 0.05, **P < 0.01, ***P < 0.001

Based on the in vivo results, to understand more fully how IL-1β impacts the TMJ disc at the cellular level and influences the disc ECM, we established an in vitro model using TMJ disc cells. We isolated primary disc cells from 4-week-old rats and confirmed that these cells exhibited the expected phenotype of fibrocartilaginous cells (Fig. 2a–c, Fig. S3), as evidenced by the expression of COL I and COL II, and positive staining with toluidine blue. We then cultured the TMJ disc cells in the presence of IL-1β to mimic inflammatory conditions and assessed proliferation using the Ki-67 expression (Fig. 2d) and cell counting kit 8 (CCK8) (Fig. 2e). Compared to the untreated controls, IL-1β significantly stimulated the proliferation of disc cells, as indicted by an increased number of Ki-67 positive nuclei.

Next, we assessed protein markers associated with collagen turnover to determine the impact of IL-1β stimulation on disc cells. Western blot analysis revealed that both COL I, an indicator of new collagen synthesis, and matrix metalloproteinase 3 (MMP3), an enzyme involved in the degradation of collagens and other ECM proteins in the TMJ disc, showed elevated expression in a dose-dependent manner after 24 h of IL-1β treatment compared to untreated controls (Fig. 2f). Interestingly, COL I expression continued to increase through 48 h, whereas MMP3 expression peaked at 12 h and then decreased from 24 h to 48 h (Fig. 2g). These results indicate that under inflammatory conditions, the balance of ECM metabolism shifts to prioritize collagen synthesis over time.

### Sustained IL-1β stimulation alters the ultrastructure and nanomechanical properties of collagen fibrils secreted by TMJ disc cells

**Fig. 3:**
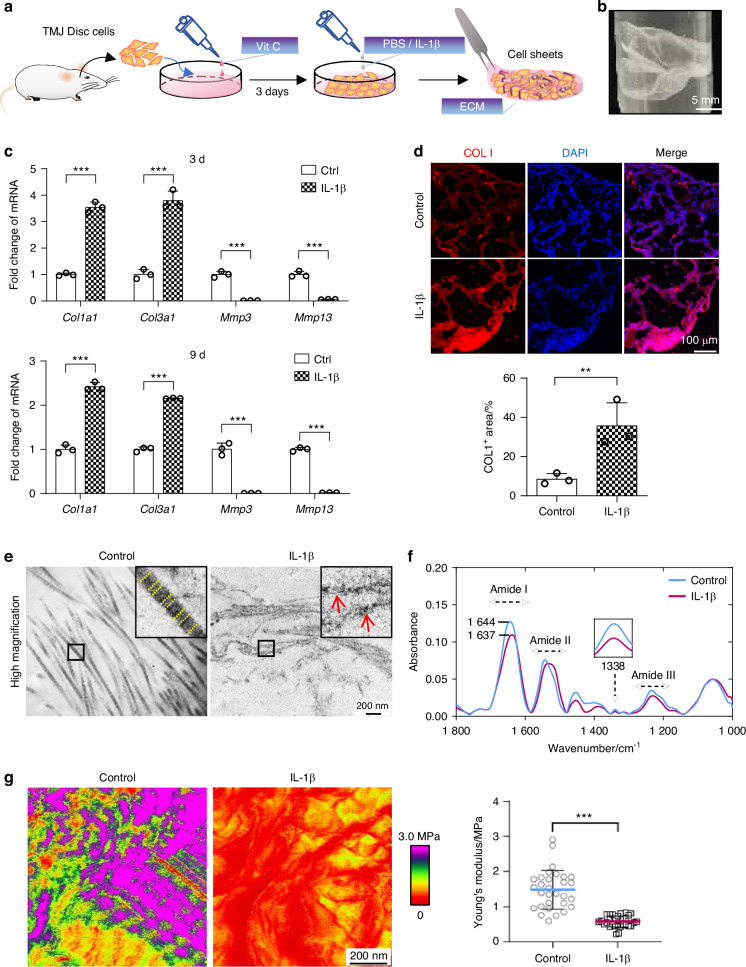
IL-1β stimulation impairs the content and nanostructure of cell-derived collagen fibrils. **a** Illustration depicting the fabrication of disc cell sheets. **b** Gross image of the disc cell sheets. **c** Expression of ECM related markers of disc cell sheets was assessed by qPCR after prolonged IL-1β treatment for 3 days (3 d) and 9 days (9 d). The results showed upregulated expression of *Col1a1* and *Col3a1*, and downregulated expression of *Mmp3* and *Mmp13*. **d** Representative immunofluorescence images and semi-quantitative statistics regarding COL I expression in cell sheets stimulated with IL-1β. **e** TEM images of ECM in cell sheets after prolonged treatment with IL-1β. Red arrows: thin microfibrils; yellow dotted line: periodic banding of collagen fibrils. **f** Representative spectra of cell sheets after baseline subtraction: the maximum peak of amide I shifted with a wide absorbance peak of amide II and weak peak of 1 338 cm ^−^^1^ after the IL-1β treatment. **g** Representative AFM nanomechanical mapping features of disc cell sheets under fluid condition after stimulated with IL-1β. The IL-1β treated group showed a significant decrease in Young’s modulus compared with the control group. **P < 0.01, ***P < 0.001

To comprehensively understand collagen assembly and remodeling in TMJ discs, we constructed a 3D cell culture model in vitro. We cultured TMJ disc cells for total 12 days to form cell sheets, allowing them to create collagen-rich ECM membranes (Fig. 3a, b). We then measured the gene expression in 3D cultured disc cell sheets of collagen metabolic markers that show sustained overexpression under inflammatory conditions, such as *Col1a1* and *Col3a1* mRNA, as well as those that appear to be repressed, such as *Mmp3* and *Mmp13* mRNA (Fig. 3c). Immunofluorescence staining showed that control disc cell sheets had a structured morphology with areas of COL I-positive staining. In contrast, COL I expression was enhanced and structurally disorganized in inflamed disc cell sheets (Fig. 3d). These results were consistent with previous findings on protein expression in 2D cultured cells.

We then examined the nanostructure of collagen fibrils produced by the cell sheets with or without IL-1β exposure via transmission electronic microscopy (TEM) (Fig. 3e). In untreated controls, we observed fibrils with periodic banding (yellow dotted line), indicative of collagen, arranged in well-organized structures. In contrast, IL-1β exposure resulted in an abundance of small and incompletely assembled microfibrils (red arrows), with a loose and disordered arrangement of fibrils (Fig. S4). We also used attenuated total reflectance Fourier transform infrared (ATR-FTIR) spectroscopy to examine the biochemical composition of the ECM networks created by the cell sheets (Fig. S5, Fig. 3f). The spectra of both control and IL-1β-treated samples exhibited collagen-characteristic absorbance peaks of amide A, B, I, II, and III bands. (Fig. S5). Among these characteristic absorption peaks, amide I (representing stretching of C = O bonds) and amide II (representing vibration in the plane of N-H bonds and C-N stretching bond) are particularly sensitive to pathological changes in collagen fibrils. To focus on these changes, we magnified the spectra between 1 000 cm^−^^1^ to 1 800 cm^−^^1^ (Fig. 3f). The control sample displayed a single sharp maximum peak at 1 644 cm^−^^1^ for amide I band, and 1 544 cm^−^^1^ for amide II band, individually. However, in the IL-1β-treated group, the amide I peak was shifted, consistent with our previous in vivo study,^16^ and a wide range of amide II maximum peak was observed. Additionally, we detected the variation of the peak at 1 338 cm^−^^1^, which corresponds to the C–H_2_ wagging vibration of proline and serves as an indicator of collagen damage.^23^ The control group performed a significant peak at 1 338 cm^−^^1^, while the peak was diminished in the IL-1β-treated group, indicating an aberrant peptide or hydrogen bonding.

We next analyzed the mechanical properties of the collagen-rich ECM networks generated from the cell sheets in a physiological condition. To achieve this, we generated modulus maps in fluids by atomic force microscopy (AFM) (Fig. 3g). The Young’s modulus of well-organized fibrils in control cell sheets was significantly higher than that of disordered ECM cell sheets treated with IL-1β. These results are consistent with our previous findings regarding the effects of persistent inflammation on TMJ discs.^16^ Taken together, these data suggest that TMJ disc cell sheets responded to long-term IL-1β stimulation by upregulating the expression of matrix-remodeling genes and profoundly altering the structure of their local collagenous matrix.

### Inflammation induces collagen remodeling in TMJ disc displacement by NF-κB activation

**Fig. 4:**
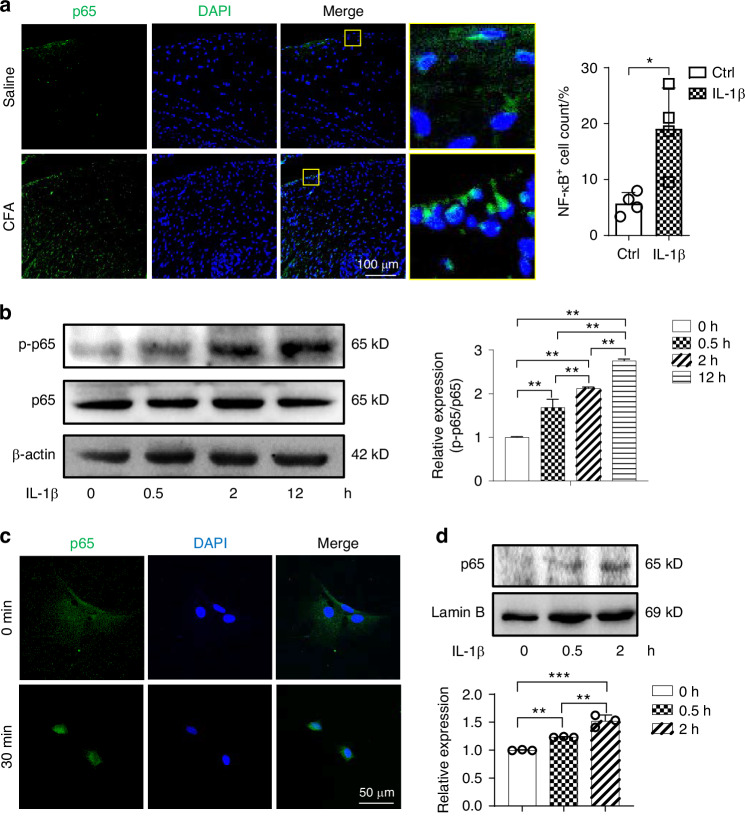
NF-κB is activated in TMJ disc cells under an inflammatory environment. **a** Representative immunofluorescence images and semi-quantitative statistics regarding NF-κB p65 staining in the posterior band of a TMJ disc at 5 week after saline/CFA 1st injection (*n* = 4). **b** The expression of p-p65 and p65 assessed via western blot. **c** Representative immunofluorescence images of p65 staining in disc cells stimulated with IL-1β for 0.5 h. **d** Expression of nuclear p65 assessed via western blot after IL-1β treatment. *P < 0.05, **P < 0.01, ***P < 0.001

To explore the possible mechanisms underlying TMJ disc ECM remodeling, we returned to the in vivo model and analyzed the expression of NF-κB p65 in control and CFA-treated rats by immunofluorescence (Fig. 4a). We observed increased NF-κB p65 staining in the inflamed discs at 5 weeks after injection, suggesting inflammation-induced NF-κB activation. Then, we assessed the phosphorylation of p65 in vitro by western blot, and found that expression of p-p65 increased after IL-1β stimulation (Fig. 4b). At the same time, the nuclear translocation of p65 was increased in the IL-1β-treated group both by immunofluorescence and western blot (Fig. 4c, d). These results indicate that NF-κB pathway is activated in TMJ disc cells under inflammatory conditions, and the abnormal collagen remodeling may be related to NF-κB activation.

**Fig. 5:**
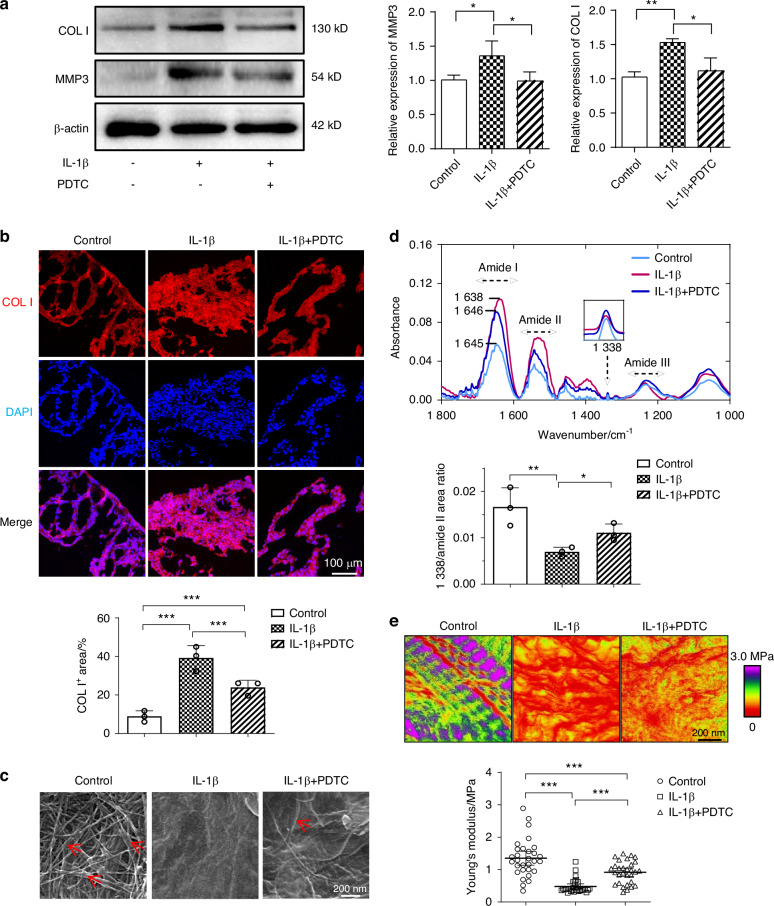
Blocking NF-κB activation partly rescues IL-1β-induced abnormal collagen remodeling and nanostructure of collagen fibrils in vitro. **a** The expression of COL I and MMP3 of 2D-cultivated disc cells was assessed by western blot. **b** Representative immunofluorescence images and semi-quantitative statistics regarding COL I in cell sheets after a NF-κB blockade and stimulation with IL-1β. **c** SEM images showing recovery of the collagen ultrastructure in cell sheets after a NF-κB blockade during prolonged exposure to an inflammatory environment. Red arrow: collagen fibrils. **d** Representative spectra of cell sheets after baseline subtraction and quantitative statistics of 1 338 cm ^−^^1^ /amide II area ratio. **e** Representative nanomechanical mapping features and non-parametric analysis of the Young’s modulus of the disc cell sheets under fluid condition. The results showed an increase in Young’s modulus after a NF-κB blockade during prolonged exposure to an inflammatory environment. *P < 0.05, **P < 0.01, ***P < 0.001

We next tested whether the collagenous matrix formed by the TMJ disc cells could be affected by modulating the NF-κB pathway. We inhibited the NF-κB pathway by using the specific inhibitor ammonium pyrrolidinedithiocarbamate (PDTC), and found COL I and MMP3 expression was significantly downregulated after 24 h IL-1β treatment in a 2D culture environment (Fig. 5a). We also assessed the structural changes of the collagenous matrix formed by the cell sheets when treated with IL-1β in the presence of PDTC (Fig. 5b, c). Immunofluorescence staining showed that the structured morphology of the disc cells in the IL-1β + PDTC group had been partly preserved, and the expression of COL I expression was reduced compared with the inflamed disc cell sheets (Fig. 5b). Scanning electronic microscopy (SEM) analysis showed well-organized collagen fibrils in the control cultures, as indicated by red arrows (Fig. 5c), suggesting the presence of collagen fibrils in normal TMJ disc cell sheets. However, after IL-1β exposure, collagen fibrils dominantly presented with sparse and amorphous structures, whereas when treated with PDTC, the structure of collagen fibrils was partly restored. The results of FTIR spectrum showed that PDTC administration reversed the inflammation related-peak shift of amide I, along with a recovered sharp peak at amide II and 1 338 cm ^−^^1^. Additionally, the area ratio of 1 338 cm^−^^1^ to amide II, which was significantly decreased in the IL-1β group, was partly recovered after PDTC administration, indicating the alleviation of collagen structural damage by blocking NF-κB (Fig. 5d). AFM scanning showed that although IL-1β treatment resulted in a significantly lower Young’s modulus, this reduction could be partly recovered by PDTC both in liquid (Fig. 5e) and air conditions (Fig. S6). Briefly, we demonstrated that blocking NF-κB under inflammatory conditions improves the structure and mechanical properties of collagen by simulating a 3D environment for ECM synthesis in vitro.

### Blocking the NF-κB pathway alleviates degenerative changes and displacement of TMJ disc in vivo

**Fig. 6:**
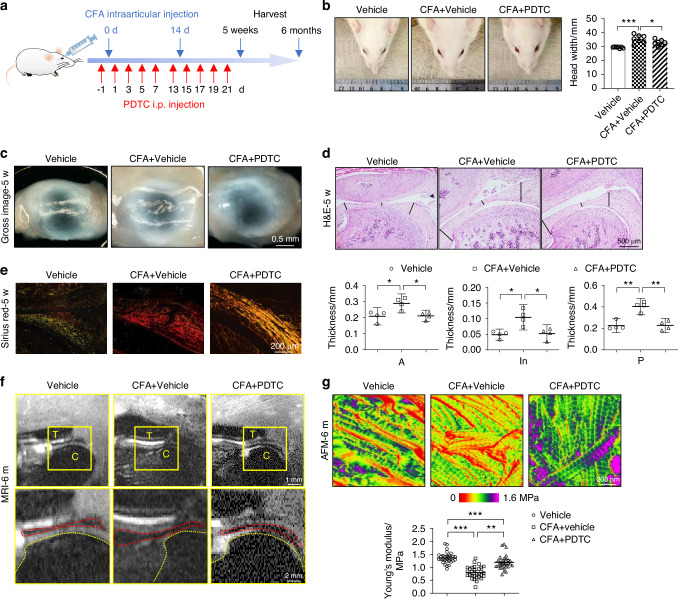
Blocking NF-κB activation alleviates the damage of collagen structure, disc deformation and displacement under inflammatory conditions in vivo. **a** Timeline of induction and PDTC treatment of chronic TMJ inflammation in rats. **b** Representative photographs and statistical analysis showing differences in head widths one day after the rats received saline/CFA injection. **c** Representative morphological features of the TMJ discs 5 weeks after PDTC administration. **d** H&E staining showing alterations in disc configuration; the statistical analysis showed differences in disc thickness among the three groups. The black lines indicate the locations where the thickness of the disc was measured (*n* = 4). A: anterior band, In: intermediate zone, P: posterior band. **e** Representative images of Sirius red staining under polarized light. **f** Representative sagittal view of T_2_ weighted-MRI images of the rat head 6 m after saline/CFA 1^st^ injection (*n* = 3): the inflamed disc is deformed, thickened at the anterior band, and the disc is dislocated; compared with the CFA+Vehicle group, the disc in the CFA + PDTC group showed regular biconcave contours, and the posterior band was opposite to the top of the condyle. T: temporal bone, C: condyle, red dotted line: contour of the disc, yellow dotted line: contour of the bone. **g** Representative nanomechanical mapping features of the posterior band in TMJ discs. Non-parametric statistical analysis showed an increase in Young’s modulus after a NF-κB blockade. *P < 0.05, **P < 0.01, ***P < 0.001

Based on the in vitro data, we again returned to the in vivo model of TMJ arthritis. We delivered PDTC intraperitoneally before and after CFA injection as Fig. 6a depicted. We observed significant swelling of TMJ area and increased head width in rats in the CFA+Vehicle group at 1 day after CFA injection (Fig. 6b). Compared with the inflammation group, the CFA + PDTC group had milder swelling and significantly smaller head width. Animals were sacrificed at 5 weeks for gross observation and histological testing or 6 months for mechanical and positional testing. The TMJ discs from the control group were semi-translucent and thin, with little synovial tissue, whereas the TMJ discs harvested from the CFA+Vehicle group were significantly thicker and appeared to be less translucent. PDTC treatment improved inflammation-induced morphological changes in the discs (Fig. 6c) and disc thickening (Fig. 6d). The intensity of collagen staining by Sirius red staining under polarized light (yellowish and red on the pictures) was increased, and the arrangement of the collagen fibers in the posterior band appeared to be more disorganized in the inflammation group, an effect that could be partly reversed by PDTC treatment (Fig. 6e). Briefly, PDTC appears to alleviate structural changes in TMJ disc matrix driven by CFA-induced inflammation.

We used MRI 6 months after the double CFA injections to assess the effect of the NF-κB blockade on the disc position and configuration. The control TMJ discs remained biconcave (Fig. 6f), whereas the inflamed discs had lost their normal configuration and showed significant swelling. Moreover, the TMJ discs in the CFA+Vehicle group were displaced anteriorly, and the boundary of the posterior band was displaced such that it was almost in front of the condyle. These findings are consistent with the known pathogenesis of the TMJ disc in TMJ disorders and resulting disc dysfunction.^24^ In contrast, the shape of the TMJ disc in the CFA + PDTC group was more normal, and the disc was properly located between the condyle and temporal bone. We also examined the mechanical properties of TMJ discs. We found that although chronic inflammation resulted in a significant decrease in Young’s modulus (Fig. 6g, Fig. S7), the measurements collected in PDTC-treated discs were significantly higher than those in the CFA+Vehicle group in both liquid and air. These findings suggest that PDTC treatment partly prevented deterioration of gross disc pathology and TMJ disc nanomechanical properties under chronic inflammatory conditions.

## Discussion

**Fig. 7:**
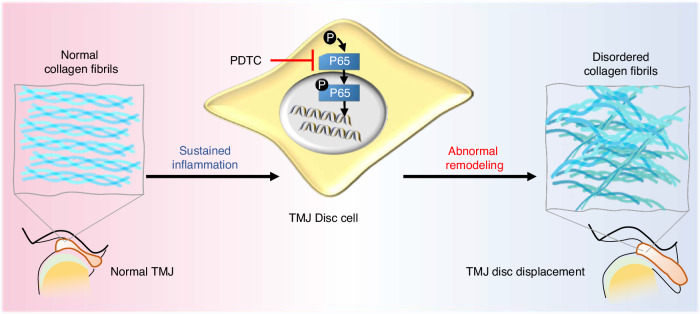
A schematic graph illustrating how sustained inflammation induces abnormal collagen remodeling and assembly via the NF-κB pathway, diminishes the mechanical properties of collagen fibrils, and aggravates degenerative changes in the collagen, ultimately resulting in disc displacement

Using a rat model, we demonstrated the pathogenic process of deformity and displacement of TMJ discs by local chronic inflammation-induced abnormal collagen remodeling. Proinflammatory cytokine IL-1β induces abnormal remodeling of the collagen matrix in TMJ disc cells, and the activation of NF-κB pathway prompt the deterioration of the ultrastructural and nanomechanical properties of collagen fibril networks. NF-κB blockade alleviated abnormal collagenous matrix remodeling and improved nanomechanical properties of inflamed TMJ discs. These results suggest that inhibiting the NF-κB pathway could attenuate the deformation and displacement of TMJ discs under sustained inflammatory conditions in vivo (Fig. 7).

IL-1β treatment was reported to induce TMJ disc cell proliferation, accompanied by enhanced expression of ECM proteins.^25^ Our results showed IL-1β simultaneously induced cell proliferation and enhanced both the synthesis and degradation of collagen at the early stages. Previous studies have demonstrated that increased collagen content and collagen-related peptides promote cell proliferation,^26–28^ suggesting that these cellular responses may be related with the altered collagen remodeling. This cell proliferation aligns with the increased cellularity in inflamed discs.^14^ Over time, a marked increase in collagen synthesis was observed, suggesting that the balance of collagen homeostasis shifted toward the synthesis side under continuous inflammatory situations,^29^ leading to spatial, morphological and functional alterations in the collagenous matrix, characteristic of the degenerative changes.

Abnormal remodeling of the collagenous matrix can negatively impact the normal function of the disc and TMJ cartilage,^30^ which is consistent with the degenerative changes observed in TMD patients.^31,32^ To mimic the physiological environment for collagen assembly, we built cell sheets from primary cultures of disc cells. With this experimental setup, we innovatively examined the structure and mechanical properties of collagen fibrils under inflammatory conditions in vitro. Compared with cell cultures in plates from previous studies, the cell sheets acquired a well-organized collagenous matrix secreted by the cultured cells.^33,34^ As the morphology of the ECM is tightly related to its function, the orientation and arrangement of the collagen fibrils contribute to the mechanical properties of the TMJ disc.^13^ Thus, cell sheets can provide a good model of the TMJ micro-environment and enable exploration of the pathogenetic mechanisms underlying degenerative joint disorders.

The main function of the disc collagenous structure is to maintain the contour and provide stress cushioning.^35^ However, based on current clinical evidence, it is difficult to uncover the comprehensive relationship between structural deterioration and disc displacement.^36,37^ Despite the increased use of MRI for clinical diagnoses of disc displacement,^38^ locating the TMJ disc in rodent models remains a challenge. Given that new methods have increased the accuracy of MRI scanning, such that it is similar to that of histological sections,^39^ we used MRI to detect changes in the morphology and position of TMJ discs in vivo. Our results demonstrate that locally sustained inflammation resulted in disc deformation, potentially contributing to the occurrence of disc displacement. However, the pathogenesis of disc displacement is multifactorial, with intra-articular mechanical stimuli playing a significant role in initiating collagen damage and promoting disc dysfunction.^40^ Further studies are needed to clarify the relationships between ECM remodeling, morphological changes and the occurrence of disc displacement.

Activation of the NF-κB pathway has been previously implicated in the pathogenesis of TMJ arthritis.^41^ In this study, we verified that NF-κB activation participates in matrix metabolism and structural degeneration following IL-1β exposure. Indeed, our results in TMJ disc cells are consistent with those studies focusing on the relationship between inflammatory cytokines and NF-κB activation in chondrocytes and synovial fibroblasts.^42,43^ Meanwhile, Xu et al. demonstrated that perforated disc cells promote angiogenesis by inducing IL-1β via NF-κB activation,^44^ supporting the regulatory function of NF-κB signaling pathway in TMJ inflammation. Specifically, we found that a NF-κB blockade partly restored the ultrastructure of extracellular collagen fibrils, which are likely to be involved in the stress cushioning of the discs. Therefore, such therapeutic effects of NF-κB blockade in disc mechanical properties might contribute to the maintenance of normal stress cushioning and could help to keep the disc in its correct location during mandibular movements. Given the lack of effective clinical approaches to reversing degeneration of the TMJ disc, our findings provide a potential approach for alleviating degenerative changes or disc dysfunction.

## Conclusion

Based on the results in the present study, chronic inflammation induces the deformity, displacement, and deteriorated mechanical properties of the TMJ discs. This inflammatory condition disrupts collagen synthesis, induces degradation and damages the ultrastructural and nanomechanical properties of extracellular collagen fibrils secreted by TMJ disc cells. The NF-κB pathway participates in the inflammation-related collagen remodeling and structural degeneration of TMJ discs. The present study provides a new understanding of the pathogenesis of TMJ disc dysfunction and offers a possible therapeutic target.

## Methods

### Animals

To evaluate the changes in inflamed TMJ discs, we obtained female 7-week-old Sprague–Dawley rats and randomly divided them into a TMJ arthritis group and a control group. TMJ arthritis was induced by a double intra-articular injection of CFA (Sigma, USA) on days 0 and 14, as previously described.^16^ The TMJ samples were harvested at 5 weeks and 6 months after the first CFA injection for MRI and histological staining.

To evaluate the role of NF-κB in collagen remodeling, we used PDTC, which is a specific inhibitor of NF-κB. Female 7-week-old Sprague–Dawley rats were randomly divided into three groups: Vehicle, CFA+Vehicle, and CFA + PDTC. PDTC (100 mg/kg, Sigma, USA) was systemically administrated via intraperitoneal (i.p.) injection on days -1, 1, 3, 5, 7, 13, 15, 17, 19, and 21,^45^ and an equivalent volume of saline was administrated as a control. The TMJ samples were harvested at 5 weeks for gross observation and histological staining or 6 months for MRI and mechanical test after the first CFA injection.

The animal procedures were approved by the Peking University Animal Ethics Committee prior to the initiation of the study (Approval number: LA2021027) and performed in accordance with the approved guidelines. To minimize potential confounders, the investigator was blinded to the animal group identity during the intra-articular and systemic injections. All rats were kept at room temperature (five rats in an individual cage) under a 12-h dark and 12-h light cycle, and had free access to food and water. All rats were sacrificed by pentobarbital overdose.

### MRI scanning

A 9.4 T MRI system (BioSpec, Bruker Biospin, Germany) was used to elucidate the location and contour of rat TMJ discs in the closed-mouth position. Rats were anesthetized by isoflurane during the process of imaging. A surface coil was placed between the rat’s bilateral external auditory meatus. The plane of the surface coil was set parallel or perpendicular to the axis of the mandibular ramus of the rats. For better image contrast under inflammatory condition, a series of rapid acquisition with relaxation enhancement sequence T2-weighted images were performed. Based on previous study,^46^ the scanning parameters were as follows: 16.44 mm × 25 mm field of view, 256 × 256 data matrix, 4 000 ms relaxation delay, 33 ms echo-time with 8 averages.

### Isolation, characterization and culture of rat TMJ disc cells

Primary TMJ disc cells were isolated from 4-week-old female rats as previously described,^47^ and cultured with Dulbecco’s modified Eagle medium (DMEM) containing 10% fetal bovine serum, 4.5 g/mL glucose, 100 U/mL penicillin-streptomycin, and 2 mmol/L L-glutamine. Disc cells were used for experiments at passage 2 to 3.

The characterization of TMJ disc cells was delivered on passage 1. The condylar chondrocytes, disc cells and fibroblast-like synoviocytes (FLS) were dissected from 4-week-old female rats. We compared the characteristic markers of fibroblasts and chondrocytes among these three types of TMJ resident cells by Toluidine Blue staining, quantitative real-time polymerase chain reaction (qPCR) and western blot analysis.

### Formation and collection of 3D cultured cell sheets

Rat primary TMJ disc cells were seeded into 6-well plates at a density of 1 × 10^6^ cells/well and cultured with DMEM containing 10% fetal bovine serum, 4.5 g/mL glucose, 50 μmol/L 2-phospho-L-ascorbic acid trisodium, 100 U/mL penicillin-streptomycin, and 2 mM L-glutamine. For collection, cell sheets were gently peeled off with tweezers from one side of the wells.

### AFM and FTIR scanning

Samples for AFM were embedded and made into frozen sections (40 μm thickness) as previously described.^16^ AFM testing was performed using an Icon atomic force microscope (Bruker, USA). The samples were scanned in PeakForce Quantitative Nanomechanics mode. ScanAsyst cantilevers (spring constant: 0.20–0.25 N•m^−1^) were utilized for cell sheets and for TMJ disc in the saline; RTESPA-150 cantilevers (spring constant: 4-5 N•m^−1^) were utilized for cell sheets and for TMJ disc in air. In order to calculate the Young’s modulus, three representative locations were scanned for each specimen. In each mapping image, ten 50 nm × 50 nm samples were randomly selected from the collagen fibril region.

For the FTIR test, cell sheets under different stimulations were collected and lyophilized for 2 h. The samples were then analyzed in ATR mode using a FTIR spectrometer (Thermo, USA). The locations of absorbance peaks of amide A, B, I, II, and III were identified using an OMNIC software, while baseline subtraction and peak area integration were performed using an Origin9 software.

### Statistical analysis

The statistical analysis was performed using SPSS software (version 20.0 for Windows). Based on the data distribution, differences in TMJ disc thickness, western blot data, qPCR data and FTIR analysis were assessed via a one-way analysis of variance with the Holm–Sidak test. All data are presented as mean ± standard deviation. Differences were analyzed by non-parametric analysis for the AFM data. *P* values < 0.05 were considered statistically significant.

The reagents used in the present study are listed in the Supplementary Table 1, and the following methods are detailed in the Supplementary materials:

- Tissue preparation and histological staining
- Immunofluorescence and immunohistochemical staining
- Treatment of TMJ disc cells
- Cell proliferation assay
- qPCR and western blot analysis
- TEM and SEM

## Supplementary information

supplementary information

## Data Availability

The authors declare that the data supporting the findings in the present study are available within the manuscript and the supplementary materials.

## References

[1] Tanaka, E. & van Eijden, T. Biomechanical behavior of the temporomandibular joint disc. *Crit. Rev. Oral. Biol. Med. : Off. Publ. Am. Assoc. Oral. Biologists* **14**, 138–150 (2003). 10.1177/154411130301400207 12764076

[2] Anastassaki Kohler, A., Hugoson, A. & Magnusson, T. Prevalence of symptoms indicative of temporomandibular disorders in adults: cross-sectional epidemiological investigations covering two decades. *Acta Odontol. Scand.* **70**, 213–223 (2012). 22126531 10.3109/00016357.2011.634832

[3] Bhargava, D., Thomas, S., Pawar, P., Jain, M. & Pathak, P. Ultrasound-guided arthrocentesis using single-puncture, double-lumen, single-barrel needle for patients with temporomandibular joint acute closed lock internal derangement. *Oral. Maxillofac. Surg.* **23**, 159–165 (2019). 30923970 10.1007/s10006-019-00753-6

[4] Bi, R. et al. Divergent chondro/osteogenic transduction laws of fibrocartilage stem cell drive temporomandibular joint osteoarthritis in growing mice. *Int J. Oral. Sci.* **15**, 36 (2023). 37626033 10.1038/s41368-023-00240-5 PMC10457315

[5] Lei, J., Yap, A. U., Liu, M. Q. & Fu, K. Y. Condylar repair and regeneration in adolescents/young adults with early-stage degenerative temporomandibular joint disease: A randomised controlled study. *J. Oral. Rehabil.* **46**, 704–714 (2019). 31009097 10.1111/joor.12805

[6] Almeida, L. E. et al. Immunohistochemical analysis of IL-1 beta in the discs of patients with temporomandibular joint dysfunction. *Cranio* **35**, 233–237 (2017). 27415587 10.1080/08869634.2016.1207911

[7] Feng, S. Y. et al. Increased chemokine RANTES in synovial fluid and its role in early-stage degenerative temporomandibular joint disease. *J. Oral. Rehabil*. **47**, 1150–1160 (2020). 10.1111/joor.13041 32609901

[8] Kellenberger, C. J. et al. Temporomandibular joint magnetic resonance imaging findings in adolescents with anterior disk displacement compared to those with juvenile idiopathic arthritis. *J. Oral. Rehabil.* **46**, 14–22 (2019). 30252949 10.1111/joor.12720

[9] Leonardi, R. et al. A histochemical survey of the human temporomandibular joint disc of patients with internal derangement without reduction. *J. Craniofac Surg.* **18**, 1429–1433 (2007). 17993895 10.1097/scs.0b013e31814fb72a

[10] Antoniadis, A. et al. Elevated secretion of pro-collagen I-alpha and vascular endothelial growth factor as biomarkers of acetabular labrum degeneration and calcification in hip osteoarthritis: An explant study. *J. Orthop. Transl.* **44**, 19–25 (2024). 10.1016/j.jot.2023.08.007 PMC10765489 38179125

[11] Trimarchi, M. et al. Mast Cell Cytokines in Acute and Chronic Gingival Tissue Inflammation: Role of IL-33 and IL-37. *Int. J. Mol. Sci.* **23**, 13242 (2022). 10.3390/ijms232113242 PMC9654575 36362030

[12] Asakawa-Tanne, Y. et al. Effects of enzymatic degradation after loading in temporomandibular joint. *J. Dent. Res.* **94**, 337–343 (2015). 25503611 10.1177/0022034514560588 PMC4438732

[13] Wright, G. J. et al. Tensile biomechanical properties of human temporomandibular joint disc: Effects of direction, region and sex. *J. Biomech.* **49**, 3762–3769 (2016). 27743627 10.1016/j.jbiomech.2016.09.033 PMC5164967

[14] Wang, X. D., Kou, X. X., Mao, J. J., Gan, Y. H. & Zhou, Y. H. Sustained inflammation induces degeneration of the temporomandibular joint. *J. Dent. Res.* **91**, 499–505 (2012). 22427270 10.1177/0022034512441946 PMC3327731

[15] Wang, X. D. et al. Deterioration of mechanical properties of discs in chronically inflamed TMJ. *J. Dent. Res.* **93**, 1170–1176 (2014). 25266714 10.1177/0022034514552825 PMC4293775

[16] Cui, S. J. et al. Chronic inflammation deteriorates structure and function of collagen fibril in rat temporomandibular joint disc. *Int J. Oral. Sci.* **11**, 2 (2019). 30783108 10.1038/s41368-018-0036-8 PMC6381164

[17] Suzuki, T., Segami, N., Nishimura, M. & Nojima, T. Co-expression of interleukin-1beta and tumor necrosis factor alpha in synovial tissues and synovial fluids of temporomandibular joint with internal derangement: comparison with histological grading of synovial inflammation. *J. Oral. Pathol. Med.* **31**, 549–557 (2002). 12269994 10.1034/j.1600-0714.2002.00022.x

[18] Alam, M. K. et al. Salivary Biomarkers and Temporomandibular Disorders: A Systematic Review conducted according to PRISMA guidelines and the Cochrane Handbook for Systematic Reviews of Interventions. *J. Oral Rehabilitation* **51**, 416–426 (2023). 10.1111/joor.13589 37731276

[19] Xue, X. T. et al. Progesterone attenuates temporomandibular joint inflammation through inhibition of NF-kappaB pathway in ovariectomized rats. *Sci. Rep.* **7**, 15334 (2017). 29127312 10.1038/s41598-017-15285-w PMC5681685

[20] Tipton, D. A., Christian, J. & Blumer, A. Effects of cranberry components on IL-1beta-stimulated production of IL-6, IL-8 and VEGF by human TMJ synovial fibroblasts. *Arch. Oral. Biol.* **68**, 88–96 (2016). 27107382 10.1016/j.archoralbio.2016.04.005

[21] Li, Y. et al. Transglutaminase 2 inhibitors attenuate osteoarthritic degeneration of TMJ-osteoarthritis by suppressing NF-kappaB activation. *Int Immunopharmacol.* **114**, 109486 (2023). 36508923 10.1016/j.intimp.2022.109486

[22] Kellesarian, S. V. et al. Cytokine profile in the synovial fluid of patients with temporomandibular joint disorders: A systematic review. *Cytokine* **77**, 98–106 (2016). 26556103 10.1016/j.cyto.2015.11.005

[23] Jackson, M., Choo, L. P., Watson, P. H., Halliday, W. C. & Mantsch, H. H. Beware of connective tissue proteins: assignment and implications of collagen absorptions in infrared spectra of human tissues. *Biochim Biophys. Acta* **1270**, 1–6 (1995). 7827129 10.1016/0925-4439(94)00056-v

[24] Zhuo, Z. & Cai, X. Y. Radiological follow-up results of untreated anterior disc displacement without reduction in adults. *Int J. Oral. Maxillofac. Surg.* **45**, 308–312 (2016). 26682646 10.1016/j.ijom.2015.11.007

[25] Kiga, N. et al. Expression of lumican and fibromodulin following interleukin-1 beta stimulation of disc cells of the human temporomandibular joint. *Eur. J. Histochem* **55**, e11 (2011). 22073367 10.4081/ejh.2011.e11 PMC3203468

[26] Chaipinyo, K., Oakes, B. W. & Van Damme, M. P. The use of debrided human articular cartilage for autologous chondrocyte implantation: maintenance of chondrocyte differentiation and proliferation in type I collagen gels. *J. Orthop. Res* **22**, 446–455 (2004). 15013108 10.1016/j.orthres.2003.07.001

[27] Hwang, S. J. et al. Human collagen alpha-2 type I stimulates collagen synthesis, wound healing, and elastin production in normal human dermal fibroblasts (HDFs). *BMB Rep.* **53**, 539–544 (2020). 32843132 10.5483/BMBRep.2020.53.10.120 PMC7607150

[28] Chu, W. C. et al. Distribution of pericellular matrix molecules in the temporomandibular joint and their chondroprotective effects against inflammation. *Int J. Oral. Sci.* **9**, 43–52 (2017). 28282029 10.1038/ijos.2016.57 PMC5379161

[29] Haneda, M. et al. Inflammatory Response of Articular Cartilage to Femoroacetabular Impingement in the Hip. *Am. J. Sports Med.* **48**, 1647–1656 (2020). 32383968 10.1177/0363546520918804 PMC8906442

[30] Fazaeli, S. et al. Alteration of structural and mechanical properties of the temporomandibular joint disc following elastase digestion. *J. Biomed. Mater Res. B Appl. Biomater* **108**, 3228–3240 (2020). 10.1002/jbm.b.34660 PMC7586824 32478918

[31] Coombs, M. C. et al. Structure-Function Relationships of Temporomandibular Retrodiscal Tissue. *J. Dent. Res.* **96**, 647–653 (2017). 28530471 10.1177/0022034517696458 PMC5444618

[32] Lu, X. L., Mow, V. C. & Guo, X. E. Proteoglycans and mechanical behavior of condylar cartilage. *J. Dent. Res.* **88**, 244–248 (2009). 19329458 10.1177/0022034508330432 PMC3317939

[33] Pillet, F., Gibot, L., Madi, M., Rols, M. P. & Dague, E. Importance of endogenous extracellular matrix in biomechanical properties of human skin model. *Biofabrication* **9**, 025017 (2017). 28493850 10.1088/1758-5090/aa6ed5

[34] Hsieh, C. F. et al. In Vitro Comparison of 2D-Cell Culture and 3D-Cell Sheets of Scleraxis-Programmed Bone Marrow Derived Mesenchymal Stem Cells to Primary Tendon Stem/Progenitor Cells for Tendon Repair. *Int. J. Mol. Sci.* **19**, 2272 (2018). 10.3390/ijms19082272 PMC6121892 30072668

[35] Betti, B. F. et al. Effect of mechanical loading on the metabolic activity of cells in the temporomandibular joint: a systematic review. *Clin. Oral. Investig.* **22**, 57–67 (2018). 28761983 10.1007/s00784-017-2189-9 PMC5748425

[36] Hirata, F. H. et al. Evaluation of TMJ articular eminence morphology and disc patterns in patients with disc displacement in MRI. *Braz. Oral. Res.* **21**, 265–271 (2007). 17710294 10.1590/s1806-83242007000300013

[37] Orhan, K., Nishiyama, H., Tadashi, S., Murakami, S. & Furukawa, S. Comparison of altered signal intensity, position, and morphology of the TMJ disc in MR images corrected for variations in surface coil sensitivity. *Oral. Surg. Oral. Med Oral. Pathol. Oral. Radio. Endod.* **101**, 515–522 (2006). 10.1016/j.tripleo.2005.04.004 16545717

[38] Roh, H. S., Kim, W., Kim, Y. K. & Lee, J. Y. Relationships between disk displacement, joint effusion, and degenerative changes of the TMJ in TMD patients based on MRI findings. *J. Cranio-Maxillo-Facial Surg. : Off. Publ. Eur. Assoc. Cranio-Maxillo-Facial Surg.* **40**, 283–286 (2012). 10.1016/j.jcms.2011.04.006 21745748

[39] Satoh, K. et al. Magnetic resonance imaging of the temporomandibular joint in the rat compared with low-powered light microscopy. *Arch. Oral. Biol.* **56**, 1382–1389 (2011). 21549351 10.1016/j.archoralbio.2011.03.024

[40] Jiang, N. et al. Microstructural, Micromechanical Atlas of the Temporomandibular Joint Disc. *J. Dent. Res.* **103**, 555–564 (2024). 38594786 10.1177/00220345241227822

[41] Wang, S., Lim, G., Mao, J., Sung, B. & Mao, J. Regulation of the trigeminal NR1 subunit expression induced by inflammation of the temporomandibular joint region in rats. *Pain* **141**, 97–103 (2009). 19058915 10.1016/j.pain.2008.10.021 PMC3491650

[42] Ahmad, N., Chen, S., Wang, W. & Kapila, S. 17beta-estradiol Induces MMP-9 and MMP-13 in TMJ Fibrochondrocytes via Estrogen Receptor alpha. *J. Dent. Res.* **97**, 1023–1030 (2018). 29621430 10.1177/0022034518767108 PMC6055253

[43] Ohta, K. et al. Differential regulation by IFNgamma on TNFalphainduced chemokine expression in synovial fibroblasts from temporomandibular joint. *Mol. Med Rep.* **16**, 6850–6857 (2017). 28901435 10.3892/mmr.2017.7432

[44] Xu, J. et al. IL-1beta-regulating angiogenic factors expression in perforated temporomandibular disk cells via NF-kappaB pathway. *J. Oral. Pathol. Med.* **45**, 605–612 (2016). 26775638 10.1111/jop.12420

[45] Karimy, J. K. et al. Inflammation-dependent cerebrospinal fluid hypersecretion by the choroid plexus epithelium in posthemorrhagic hydrocephalus. *Nat. Med.* **23**, 997–1003 (2017). 28692063 10.1038/nm.4361

[46] Shafqat, Q. et al. Acute Dilation of Venous Sinuses in Animal Models of Mild Traumatic Brain Injury Detected Using 9.4T MRI. *Front Neurol.* **11**, 307 (2020). 32411081 10.3389/fneur.2020.00307 PMC7198763

[47] Kapila, S., Lee, C. & Richards, D. W. Characterization and identification of proteinases and proteinase inhibitors synthesized by temporomandibular joint disc cells. *J. Dent. Res.* **74**, 1328–1336 (1995). 7629341 10.1177/00220345950740061301

