# Supplementary material for: Inflammation-related collagen fibril destruction contributes to temporomandibular joint disc displacement via NF-κB activation
Source: Int J Oral Sci. 2025 Apr 17;17:35. doi: 10.1038/s41368-025-00352-0 (PMC12006360; doi:10.1038/s41368-025-00352-0)
Supplement: Supplementary file 1 — supplementary information [file 41368_2025_352_MOESM1_ESM.docx]

**Inflammation-related collagen fibril destruction contributes to temporomandibular joint disc displacement via NF-κB activation**

Shengjie Cui, Yanning Guo, Yu Fu, Ting Zhang, Jieni Zhang, Yehua Gan, Yanheng Zhou, Yan Gu, Eileen Gentleman, Yan Liu*, Xuedong Wang*.

*Correspondence to: [orthoyan@bjmu.edu.cn](mailto:orthoyan@bjmu.edu.cn) (Y. L.) and [wangxuedong@bjmu.edu.cn](mailto:wangxuedong@bjmu.edu.cn) (X. W.)

**This file includes:**

Supplementary materials and methods

Tables S1 and S2

Figures S1 to S7

**Supplementary materials & methods**

**Tissue preparation and histological staining**

Entire temporomandibular joints (TMJs) were removed, fixed in 4% paraformaldehyde in 0.1 mol/L phosphate-buffered saline and demineralized in 10% ethylenediaminetetraacetic acid. The specimens were dehydrated in graded alcohol and xylene, embedded in paraffin, and sectioned to 5 μm thickness. The sections were stained with hematoxylin and eosin (H&E) and Sirius red staining. The thickness of anterior band, intermediate zone, and posterior band of TMJ discs was measured as previously described ^1^.

**Immunofluorescence and immunohistochemical staining**

For immunofluorescence, Disc cells were fixed in 4% paraformaldehyde, then washed in phosphate buffer saline. Cells or sections were incubated with primary antibodies of anti-COL I (1:200, Santa-Cruz, USA), Ki-67 (1:50, Abcam, USA), TNF-α (1:50, Abcam, USA), and NF-κB p65 (1:100, Cell Signaling Technology). After incubated overnight, cells or sections were washed and stained with fluorescein isothiocyanate (FITC) or tetramethylrhodamine isothiocyanate (TRITC)-conjugated secondary antibodies (1:200, Jackson Immuno Research Laboratories, West Grove, PA). Nuclei were stained with 4',6-diamidino-2-phenylindole (DAPI). Confocal microscopic images were obtained using a laser Zeiss LSM 510 scanning microscope (Jena, Germany), and processed through LSM 5 Release 4.2 software.

For immunohistochemical staining, the sections were blocked with 5% bovine serum albumin and incubated with primary antibodies against IL-1β (1:200, Santa Cruz Biotechnology) at 4°C overnight. After washing with phosphate-buffered saline (PBS), the sections were incubated with horseradish peroxidase (HRP)-conjugated secondary antibodies and the staining was revealed with diaminobenzidine.

**Treatment of TMJ disc cell cultures**

To deliver inflammatory stimulus, IL-1β at 20 ng/ml was added to the culture medium for time courses of 0 h, 2 h, 12 h, 24 h, and 48 h; for dose-response test, IL-1β was added to the culture medium for 24 h at concentrations of 0, 5, or 20 ng/ml. For nuclear translocation test, IL-1β was added to the culture medium at a concentration of 20 ng/ml for 0, 0.5, or 2 h. For cell sheets, IL-1β was added at a concentration of 20 ng/ml to culture medium at day 3, and persisted until the cell sheets were collected on day 12.

To block the NF-κB pathway, the specific inhibitor PDTC (25 μM) (Sigma, USA) was added to culture medium 1 h prior to inflammatory stimulation.

**Cell proliferation assay**

Disc cells were inoculated into 96-well plates as the density of 1×10^4^ /well. After treated with IL-1β for 24 h, CCK8 was added into the wells and O.D. values were recorded at 450 nm wavenumber.

**Quantitative real-time polymerase chain reaction (qPCR) Analysis**

Total RNA was isolated from disc cells by TRIzol reagent (Invitrogen, USA). RNA samples were reverse-transcribed using an cDNA synthesis kit (TAKARA, Japan). Quantitative Real-time PCR detection was performed on a 7500 real-time PCR System (Applied Biosystems) using SYBR Green (Roche, USA). The primers used were listed in supplementary table 2.

**Western blot**

Total proteins of primary disc cells were washed in phosphate buffer saline and lysed using a protein extraction kit (RIPA Cocktail, Thermo). Nuclear proteins were extracted using a Nuclear-Cytosol Extraction Kit (Solarbio, China). Equal amounts of proteins were separated by SDS-PAGE and then transferred onto a polyvinylidene difluoride membrane. The samples were blocked with 5% non-fat milk for 1 h, and then incubated with anti-COL I (1:1000, Proteintech, USA), MMP3 (1:1000, Abcam), NF-κB p65 and p- NF-κB p65 (1:1000, Cell Signaling Technology), β-actin (1:5000, ZSGB-BIO), COL II (1:500, Santa Cruz Biotechnology), Vimentin (1:1000, Santa Cruz Biotechnology), Lamin B (1:1000, Bioss) primary antibodies overnight at 4°C. The samples were detected using an HRP-conjugated secondary antibody.

**Transmission electron microscopy (TEM) and Scanning electron microscopy (SEM) test**

For morphological observation by TEM, cell sheets were fixed with 2.5% glutaraldehyde solution. The samples were embedded in epoxy resin. Before observed by TEM at 120 kV (FEI Tecnai Spirit, USA), ultrathin sections (70 nm) were stained with uranyl acetate and lead citrate.

To estimate collagen fibers of cell sheets, the samples were collected, and directly critical point dried to tear cells apart from the ECM. The samples were rinsed by double distilled water to remove cell debris and dehydrated. The superficial morphology of cell sheets was observed under SEM at 10 kV (Quanta FEG 650, USA).

**Table S1. List of** **reagents used in the present study**

| **Chemicals and recombined proteins** | | |
| --- | --- | --- |
| Penicillin-Streptomycin | Gibco | #15140-122 |
| Trypsin-EDTA | Gibco | #25200056 |
| L-Glutamine | Gibco | #25030081 |
| Fetal bovine serum | Hyclone | #SH30406.05 |
| TRIzol | Invitrogen | #15596026 |
| Recombinant Rat IL-1β | Peprotech | #400-01B |
| SYBR Green | Roche | #4913914001 |
| 2-phospho-L-ascorbic acid trisodium | Sigma-Aldrich | #49752 |
| PDTC | Sigma-Aldrich | #P8765 |
| Toluidin blue solution | Solarbio | #G3663 |
| Nuclear-Cytosol Extraction Kit | Solarbio | #EX1470 |
| PrimeScript RT Master Mix | TAKARA | #RR036A |
| RIPA | Thermo Scientific | #89900 |
| Halt protease inhibitor cocktail | Thermo Scientific | #78429 |
| **Primary Antibodies** | | |
| Anti-MMP3 | Abcam | #ab53015 |
| Anti-Ki-67 | Abcam | #ab15580 |
| Anti-TNF-α | Abcam | #ab1793 |
| Anti-Lamin B | Bioss | #bs-55118R |
| Anti-phospho-NF-κB p65 | Cell Signaling Technology | #3033 |
| Anti-NF-κB p65 | Cell Signaling Technology | #6956 |
| Anti-Collagen I | Proteintech | #14695-1-AP |
| Anti-β-actin | ZSGB-BIO | #TA-09 |
| Anti-Collagen I | Santa-Cruz Biotechnology | #sc-59772 |
| Anti-Collagen II | Santa-Cruz Biotechnology | #sc-52658 |
| Anti-Vimentin | Santa-Cruz Biotechnology | #sc-6260 |
| Anti-IL-1β | Santa-Cruz Biotechnology | #sc-52012 |

**Table S2. Primers used in the present study**

| **Gene** | **Forward (5’-3’)** | **Forward (5’-3’)** |
| --- | --- | --- |
| *β-actin* | TGACAGGATGCAGAAGGAGA | TAGAGCCACCAATCCACACA |
| *Mmp3* | ACCTATTCCTGGTTGCTG | GGTCTGTGGAGGACTTGTA |
| *Mmp13* | CTGACCTGGGATTTCCAAAA | ACACGTGGTTCCCTGAGAAG |
| *Col1a1* | TGCAAGAACAGCGTAGCC | CAGCCATCCACAAGCGT |
| *Col3a1* | GAAAAAACCCTGCTCGGAATT | GGATCAACCCAGTATTCTCCACTCT |


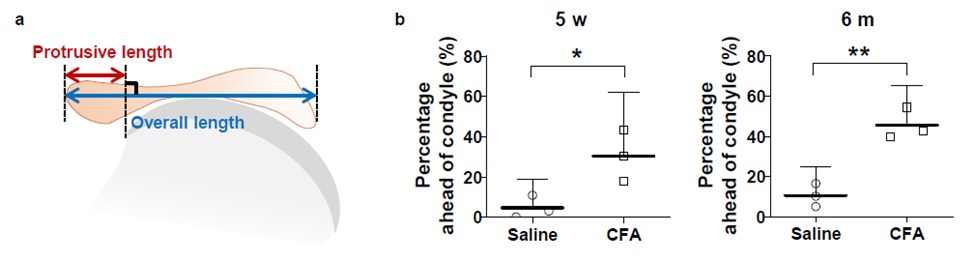
**Supplementary Figures**


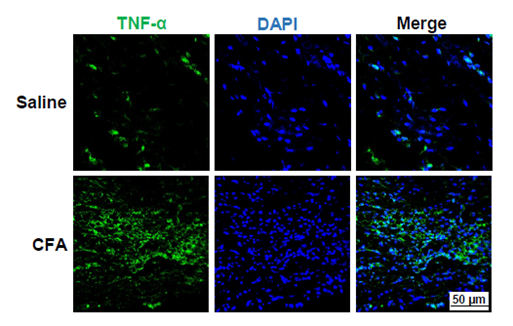
**Fig. S1** The methods and semi-quantitative analysis of the disc location in MRI test. (a) the diagram of the definition of disc overall length and the protrusive length ahead of the condyle. (b) the results of percentage of TMJ disc ahead the condyle. **P* < 0.05. ***P* < 0.01.

**Fig. S2** Representative images of immunohistochemical staining of TNF-α of normal and inflamed synovial tissue at 5 weeks after 1^st^ CFA injection.


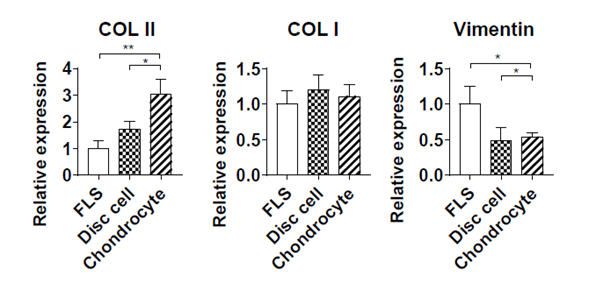
**Fig. S3** The semi-quantitative analysis of western blot results for TMJ disc cell identification. **P* < 0.05. ***P* < 0.01.


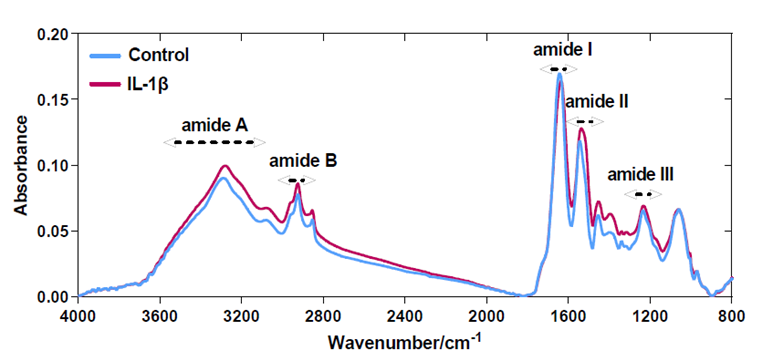

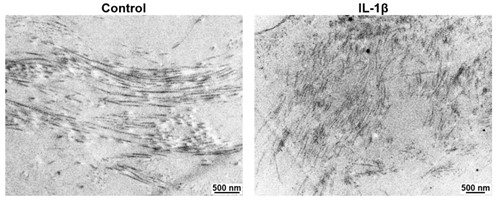
**Fig. S4** TEM images of ECM in cell sheets with lower magnification after prolonged treatment with IL-1β.

**Fig. S5** Raw spectra of ATR-FTIR without baseline subtraction of the control and IL-1β-treated cell sheets. The range of amide bands were labeled.


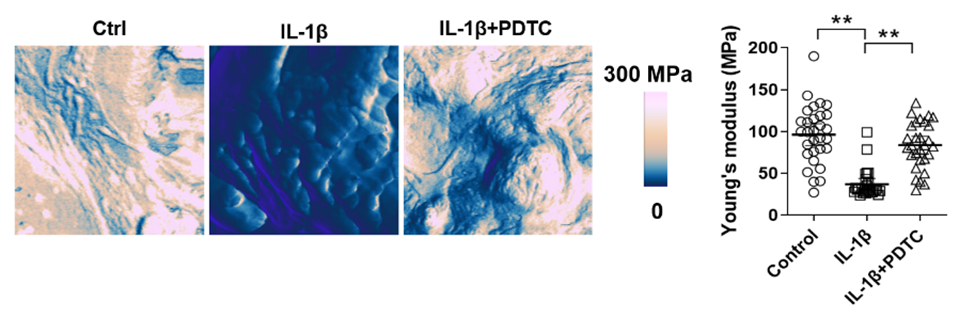


**Fig. S6** Representative nanomechanical mapping features and non-parametric analysis of the Young’s modulus of the disc cell sheets in air. The results showed an increase in Young’s modulus after a NF-κB blockade during prolonged exposure to an inflammatory environment. ***P* < 0.01.


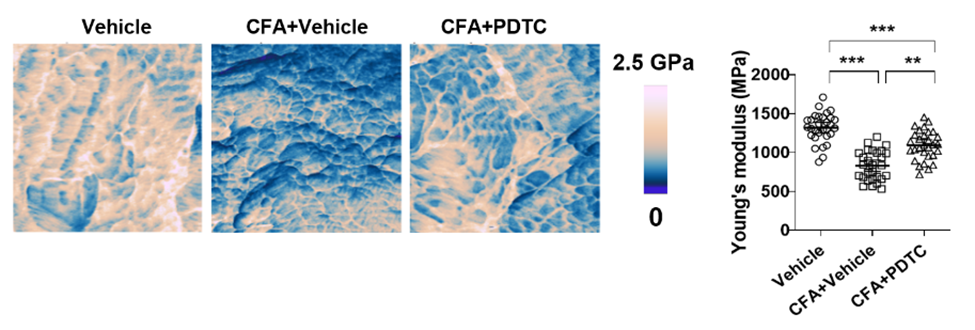


**Fig. S7** Representative nanomechanical mapping features of the posterior band in TMJ discs in air. Non-parametric statistical analysis showed an increase in Young’s modulus after a NF-κB blockade. ***P* < 0.01, ****P* < 0.001.

**References**

1 Wang, X. D., Kou, X. X., Mao, J. J., Gan, Y. H. & Zhou, Y. H. Sustained inflammation induces degeneration of the temporomandibular joint. *Journal of dental research* **91**, 499-505 (2012). <https://doi.org:10.1177/0022034512441946>
